# Automatic Classification of Screen Gaze and Dialogue in Doctor-Patient-Computer Interactions: Computational Ethnography Algorithm Development and Validation

**DOI:** 10.2196/25218

**Published:** 2021-05-10

**Authors:** Samar Helou, Victoria Abou-Khalil, Riccardo Iacobucci, Elie El Helou, Ken Kiyono

**Affiliations:** 1 Global Center for Medical Engineering and Informatics Osaka University Osaka Japan; 2 Academic Center for Computing and Media Studies Kyoto University Kyoto Japan; 3 Department of Urban Management, Graduate School of Engineering Kyoto University Kyoto Japan; 4 Faculty of Medicine Saint Joseph University Beirut Lebanon; 5 Graduate School of Engineering Science Osaka University Osaka Japan

**Keywords:** computational ethnography, patient-physician communication, doctor-patient-computer interaction, electronic medical records, pose estimation, gaze, voice activity, dialogue, clinic layout

## Abstract

**Background:**

The study of doctor-patient-computer interactions is a key research area for examining doctor-patient relationships; however, studying these interactions is costly and obtrusive as researchers usually set up complex mechanisms or intrude on consultations to collect, then manually analyze the data.

**Objective:**

We aimed to facilitate human-computer and human-human interaction research in clinics by providing a computational ethnography tool: an unobtrusive automatic classifier of screen gaze and dialogue combinations in doctor-patient-computer interactions.

**Methods:**

The classifier’s input is video taken by doctors using their computers' internal camera and microphone. By estimating the key points of the doctor's face and the presence of voice activity, we estimate the type of interaction that is taking place. The classification output of each video segment is 1 of 4 interaction classes: (1) screen gaze and dialogue, wherein the doctor is gazing at the computer screen while conversing with the patient; (2) dialogue, wherein the doctor is gazing away from the computer screen while conversing with the patient; (3) screen gaze, wherein the doctor is gazing at the computer screen without conversing with the patient; and (4) other, wherein no screen gaze or dialogue are detected. We evaluated the classifier using 30 minutes of video provided by 5 doctors simulating consultations in their clinics both in semi- and fully inclusive layouts.

**Results:**

The classifier achieved an overall accuracy of 0.83, a performance similar to that of a human coder. Similar to the human coder, the classifier was more accurate in fully inclusive layouts than in semi-inclusive layouts.

**Conclusions:**

The proposed classifier can be used by researchers, care providers, designers, medical educators, and others who are interested in exploring and answering questions related to screen gaze and dialogue in doctor-patient-computer interactions.

## Introduction

### Background

Doctor-patient communication is a combination of verbal and nonverbal expressions and can affect patient satisfaction, adherence, disclosure, and outcomes [1-8]. Health communication researchers have examined various aspects of clinician-patient verbal interactions, such as the content of the clinician’s speech and their voice tone [5,9] and the intent that an utterance has in communication [10]. Various nonverbal aspects have also been examined, such as facial expressions, eye contact, body posture, fluency [5,11], and the physical distance between clinicians and patients [12]. With the widespread adoption of electronic medical record systems, computers have become an integral part of clinics. As a result, the traditional 2-way doctor-patient relationship has been replaced by a triadic relationship among doctor, patient, and computer [13]. The use of electronic medical record systems during consultations has been shown to affect doctor-patient verbal [14] and nonverbal [2] communication, and consequently, doctor-patient relationships both positively and negatively [15,16]. Accordingly, the study of doctor-patient-computer interactions has become a key research area for examining doctor-patient relationships [17].

### Doctor-Patient-Computer Interactions

Multiple studies, mainly in primary care settings, noted that doctor-patient communication is affected [18-25] and even shaped [26] by the use of computers during clinical encounters. The use of computers was shown to modify or amplify doctors’ verbal and nonverbal behaviors [16,21,27-29] that are essential to avoid communication failures and to have effective doctor-patient communication [20]. Examples of negative verbal and nonverbal behaviors that could be amplified by the use of a computer include lack of eye contact, deficient active listening, avoidance, and interruption [30-32].

In addition to studying the effect of computer use on doctor-patient interactions, multiple studies [25,33] examined factors that affect the way these computers are used. Pearce et al [34] described the overarching styles and behaviors of doctors, patients, and computers by studying the orientation of the general practitioners' and patients' bodies as well as their conversations. Chan et al [35] found that doctors spent 50% less time using computers in examinations with psychological components than in examinations with no psychological components. Lanier et al [36] found that consultation content, physicians’ gender and level of experience, and whether the consultation was new or a follow-up were modestly related to the way physicians used the computer in primary care settings.

### Computational Ethnography Inside Clinics

Researchers studying doctor-patient-computer interactions need to identify which interactions are taking place during the consultations. To do so, researchers have used qualitative methods such as taking notes during live observations [31,37], conducting interviews [37,38], administering questionnaires [39], and sending unannounced standardized patients to collect information [40] and quantitative methods such as videotaping consultations and manually coding the videos [36,41,42] or setting up complex mechanisms for automatic data collection and analysis inside the clinics [43]. Methods that include direct observations are likely to generate more accurate data than clinician or patient reports; however, direct observations are costly in terms of time and human resources, may be obtrusive in a clinical environment, and may cause the participants to knowingly or unknowingly alter their behavior (because of the presence of an observer) [44]. Moreover, they present privacy and ethical concerns for patients and doctors such as concerns about data security and anonymization; changes to the research question that make it different from the one described in initial consent forms, and researchers’ inability to take into account all nonpublic information or situations that will be accessed [45].

Given recent technological advancements, computational ethnography has been proposed as an alternative method for studying doctor-patient-computer interaction in depth. Computational ethnography was defined as a new family of methods for conducting human-computer interaction studies in health care settings by using “automated and less obtrusive (or unobtrusive) means for collecting in situ data reflective of real end users’ actual, unaltered behaviors using a software system or a device in real-world settings [46].”

Recently, a number of tools that automate the measurement and analysis of specific behaviors in clinical settings were proposed and evaluated: Hart et al. [47] proposed and validated an automated video analysis tool to measure the synchrony and dominance in doctor-patient interactions by analyzing the cross-correlation of the kinetic energy and the frequency spectrum of their motion [47]. Gutstein et al reported developing a system that automatically learns the physician's gaze using their hand positioning [48] or body positioning and optical flow [49]. Weibel et al [43] introduced a solution that enables the capture of multimodal activity in clinical settings [43] to support computational ethnography studies in clinics. Their solution combined computer logging functionality, body motion tracking, audio detection, and eye tracking. By synchronizing data from these sensors, Weibel et al [43] were able to detect the person talking, whether the doctor is looking at the screen, the amount of gesturing, the cognitive load, information searching behavior, workflow interruptions, and the amount of computer activity; however, their solution had some limitations. First, the accuracy of the automatic classification was not reported. Second, they noted that the use of Kinect presents some limitations such as the need to set up the machine, Kinect’s inability to reidentify a body once it re-enters the scene, and the occasional transfer of skeletal tracking from human to nonhuman objects. Third, to detect the person who was talking, a Dev-Audio Microcone [50] was used. This means that such a tool may not fit the needs of people looking for a cheap and portable solution with a known robustness level. In this case, recent advancements in pose and voice activity detection algorithms could address some of these limitations. For video consultations, Faucett et al [51] created ReflectLive, a tool that provides real-time feedback to clinicians about speaking contributions, interruptions, eye gaze, and face position. ReflectLive [51] uses an open-source library for audio analysis and a commercial Javascript-based computer-vision face-tracking software for visual analysis. The real-time feedback provided by ReflectLive was evaluated in terms of its usefulness to the clinicians, but the feedback’s accuracy was not reported.

Currently, there are few truly robust and unobtrusive computational ethnography tools for clinical settings, as most tools require researchers to add external artifacts into the clinical environment. Moreover, to our knowledge, none of the existing tools is freely available to the public. To enable human-computer and human-human interaction studies in clinical settings, there is a need for publicly available, robust, unobtrusive, and automated tools for detecting and classifying doctor-patient-computer interactions.

### Aims

We aimed to provide a public, robust, unobtrusive, privacy-ensuring, and automated tool for detecting and classifying screen gaze and dialogue in doctor-patient-computer interactions. We chose to focus on screen gaze and dialogue due to recent advancements in machine learning that render the automatic and accurate estimation of pose and voice activity possible.

## Methods

### Overview

**Figure 1:**
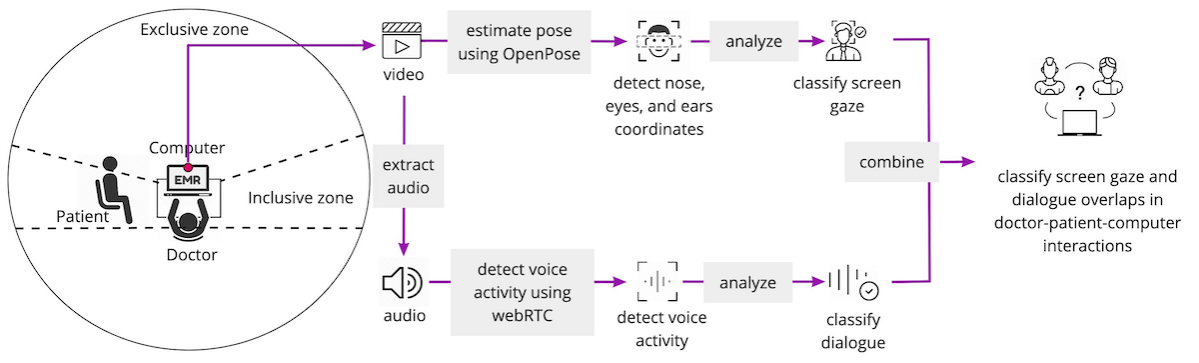
Overview of the classification process. EMR: electronic medical record.

The purpose of the classifier (Figure 1) was to detect the following interactions: (1) screen gaze and dialogue: doctor gazing at the computer screen while having a conversation with the patient; (2) dialogue: doctor conversing with the patient while looking away from the computer screen, or (3) screen gaze: doctor gazing at the computer screen without conversing with the patient. Any other type of interaction in which the doctor and the patient were not having a conversation and the doctor is not gazing at their computer screen were considered out of scope.

The code of the proposed classifier is publicly available [52] and can be used by researchers, care providers, designers, medical educators, and others who are interested in exploring and answering questions related to screen gaze and dialogue combinations in doctor-patient-computer interactions.

### Screen Gaze Classifier

The purpose of the screen gaze classifier was to detect when the doctor's gaze was aimed at the computer screen. The input of the classifier was the video captured by the doctor's computer camera and the output was a binary classification: *no screen gaze* or *screen gaze*.

**Figure 2:**
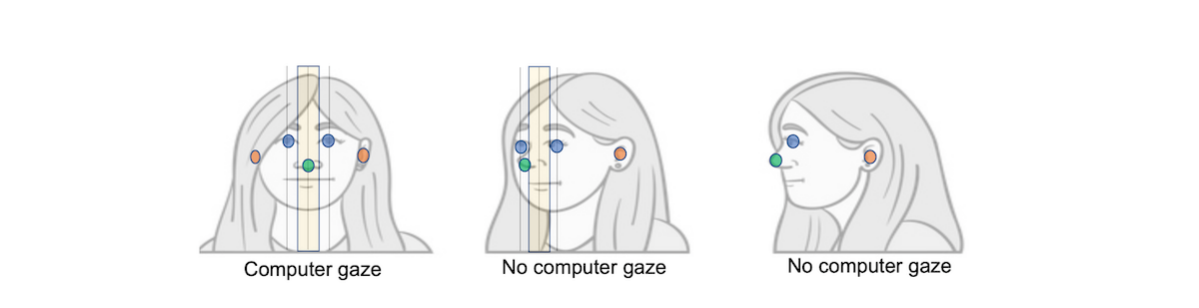
Classifying the doctor's computer screen gaze using face key point estimation.

We used the pose estimation library OpenPose [53] as a tool to detect the coordinates of key points of the doctor's face. OpenPose is an open-source library that allows real-time multiperson key point detection for body, face, hands, and feet. We extracted the coordinates of the doctor's eyes (*x*_LeftEye_, *y*_LeftEye_), (*x*_RightEye_, *y*_RightEye_), ears (*x*_LeftEar_, *y*_LeftEar_), (*x*_RightEar_, *y*_RightEar_), and nose (*x*_Nose_, *y*_Nose_), and using the coordinates, we assumed that the doctor's gaze was targeting the computer screen if (1) the location of both the doctor's ears could be estimated, and (2) the doctor's nose was centered between the eyes. For the second condition, we allowed a tolerance equal to half the distance between the 2 eyes. We assessed these criteria (Figure 2) using the following equations for each frame in the video:


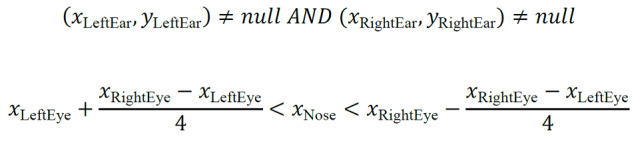


Then we assigned to each 0.5-second interval of video the most frequent classification of its corresponding frames. This results in a binary classification (no screen gaze, screen gaze) for each 0.5 seconds of video.

### Dialogue Classifier

The purpose of the dialogue classifier was to detect when the doctor and patient were engaging in conversation. The input of the classifier was the audio captured by the doctor's computer’s microphone, and the output was a binary classification of the doctor-patient conversation: *no dialogue* or *dialogue*.

We used a library based on the webRTC voice activity detection engine (an open source project maintained by the Google WebRTC team [54]). The voice activity detection library allows the detection of voice activity in an audio file by processing audio segments and estimating the probability in each segment.

We set the length of each audio segment to 5 milliseconds, which we found to offer the best results through trial and error. We set the voice activity detection to its highest aggressiveness mode in order to increase the probability of filtering out nonspeech. We assigned to each 0.5-second interval of audio the most frequent classification of its corresponding segments. This results in a binary classification (no dialogue, dialogue) for each 0.5 seconds of audio.

### Classifier of Screen Gaze and Dialogue Combinations

By combining the results of the screen gaze classifier and the dialogue classifier described above, we classify doctor-patient-computer interactions into 4 different classes (Table 1). The Screen Gaze and Dialogue (SG+D) class defines interactions wherein the doctor is gazing at the computer screen while conversing with the patient. The Dialogue (D) class defines interactions wherein the doctor is looking away from the computer screen and conversing with the patient, and the Screen Gaze (SG) class defines interactions where the doctor is gazing at the computer screen and not conversing with the patient. An interaction wherein the doctor is neither looking at the computer screen nor conversing with the patient is classified as Other. For each 0.5 seconds of video, the interactions classifier assigns 1 of the 4 classes.

**Table 1:** Four classes of doctor-patient-computer interactions.

Components			Class	
Screen gaze	Dialogue	Doctor-Patient-Computer interaction		Label
Screen gaze	Dialogue	Screen Gaze + Dialogue		SG+D
No screen gaze	Dialogue	Dialogue		D
Screen gaze	No dialogue	Screen Gaze		SG
No screen gaze	No dialogue	Other		Other

### Evaluation of the Classifier

**Figure 3:**
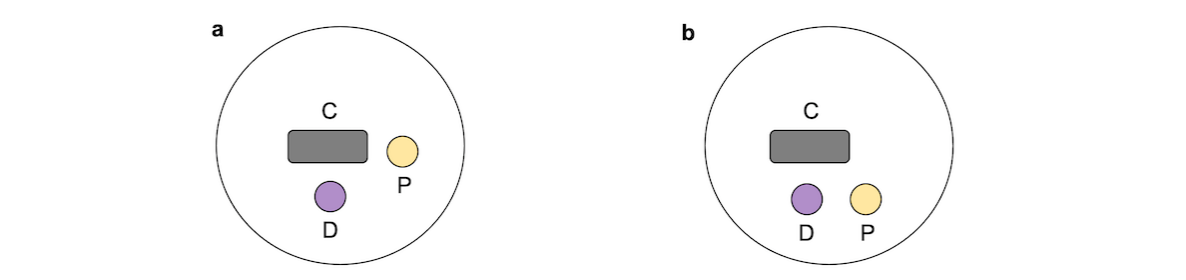
(a) Semi-inclusive and (b) fully inclusive layouts were considered in the evaluation. C: computer; D: doctor; P: patient.

We considered 2 clinical layouts in our evaluation: a semi-inclusive layout, where the patient is seated next to the computer desk, and a fully inclusive layout, where the patient is seated next to the doctor and facing the computer desk (Figure 3). The data that we used to evaluate our classifier consisted of 10 videos provided by 5 physicians. Each physician provided 2 videos—1 video simulating a consultation in a fully inclusive layout and 1 video simulating a consultation in a semi-inclusive layout. Each video was approximately 3 minutes long.

For ground truth data, each video was initially annotated by a human coder. The coder assigned 1 of the 4 interaction classes to each 0.5 seconds of video. The coder reviewed the videos several times and refined the initial annotations until they were satisfied.

To evaluate the classifier, we compared the classifier’s performance to that of a different human coder. This second coder was allowed to go through the video only once. This was to simulate a real-world scenario of video coding assigned to an external coder. The performance of the classifier and that of the second human coder were assessed in relation to the ground truth data (generated by the first coder).

The overall performance reflects the performance over the 10 videos including 5 videos in a semi-inclusive layout and 5 videos in a fully inclusive layout. The performances were assessed using an overall accuracy measure in addition to measures of precision, recall, F1 scores for each class. For each class, the support number (ie, the number of its occurrences in the ground truth data set) is reported. Weighted scores of precision, recall, and F1 scores, where the weight of a class is proportional to its support, were also measured. Difference in performance between the classifier and the human coder were assessed using 2-tailed independent *t* tests with *P* values <.05 considered statistically significant. We first report the overall performance, which reflects the performance over the 10 videos. Then, we separately report the performances over the 5 videos in a semi-inclusive layout and the 5 videos in a fully inclusive layout.

## Results

### Overall Performance

Table 2 shows the overall performances of the classifier and the human coder. The classifier showed a slightly lower overall accuracy than the coder (classifier: 0.83; human coder: 0.85); however, there was no significant difference between the accuracy of the classifier and that of the human coder (t_18_=0.6, *P*=.55).

The F1 scores of both the classifier and the coder were better when classifying SG+D (classifier: 0.81; human coder: 0.81) and D (classifier: 0.89; human coder: 0.90) than that when classifying SG (classifier: 0.63; human coder: 0.55) and Other (classifier: 0.35; human coder: 0.36) interactions. Since the D class and the SG+D class were the most frequent interactions (D: 2415/3921, 62%; SG+D: 1189/3921, 30%), the overall accuracies mainly reflect performances for these 2 classes.

**Figure 4:**
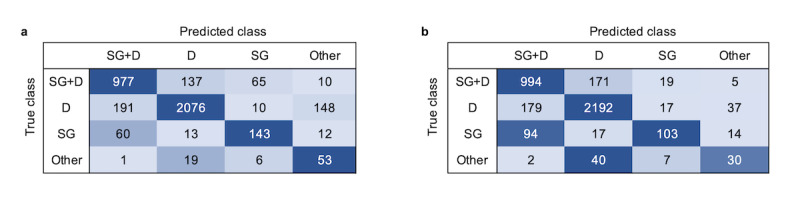
(a) Classifier and (b) human coder confusion matrices for overall performance. D: Dialogue; SG: Screen Gaze; SG+D: Screen Gaze + Dialogue.

Confusion matrices for overall performance (Figure 4) show that the classifier and the coder had similar patterns. Both mistook SG+D for D and vice versa, SG for SG+D, and Other for D interactions. The main difference between the classifier and the coder is that the classifier tended to mistake D for Other interactions, whereas the coder tended not to.

**Table 2:** Overall performance of the classifier.

Classes		Classifier					Human coder					Support, n
		Precision	Recall	F1 score	Accuracy	Precision		Recall	F1 score	Accuracy		
**All**		—^a^	—	—	0.83	—		—	—	0.85	—	
	SG+D^b^	0.79	0.82	0.81	—	0.78		0.84	0.81	—	1189	
	D^c^	0.92	0.86	0.89	—	0.91		0.90	0.90	—	2425	
	SG^d^	0.64	0.63	0.63	—	0.71		0.45	0.55	—	228	
	Other	0.24	0.67	0.35	—	0.35		0.38	0.36	—	79	
Weighted score		0.85	0.83	0.84	—	0.85		0.85	0.84	—	—	

^a^Not calculated or not applicable.

^b^SG+D: Screen Gaze and Dialogue.

^c^D: Dialogue.

^d^SG: Screen Gaze.

### Performance in a Semi-inclusive Layout

Table 3 shows the performances of the classifier and human coder for a semi-inclusive layout. The classifier had a slightly lower accuracy than the coder (classifier: 0.80; human coder: 0.83); however, there was no significant difference between the accuracy of the classifier and that of the human coder in the semi-inclusive layout (*t*_8_=1.04, *P*=0.32).

Both the classifier and the coder performed well when classifying D (classifier: F1 score 0.86; human coder: F1 score 0.88) and SG+D (classifier: 0.79; human coder: 0.82) interactions. The classifier had a slightly better F1 score than the coder when detecting SG (classifier: 0.47; human coder: 0.45), but both the classifier and the coder had low F1 scores when classifying Other interactions (classifier: 0.24; human coder: 0.21). The D and the SG+D classes had the most support (D: 1157/1958, 59%; SG+D: 702/1958, 36%) thus the overall accuracies mainly reflect performances for these 2 classes.

**Figure 5:**
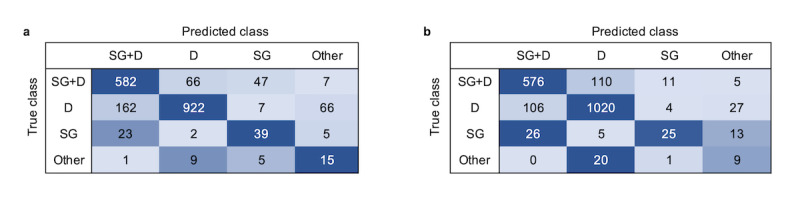
(a) Classifier and (b) human coder confusion matrices for semi-inclusive layout. D: Dialogue; SG: Screen Gaze; SG+D: Screen Gaze + Dialogue.

Confusion matrices of the classifier and the human coder (Figure 5) for a semi-inclusive layout show somewhat similar patterns for the classifier and the coder. Both mostly mistook D for SG+D and vice versa. The classifier tended to mainly mistake SG for SG+D, whereas the coder mistook SG for Other interactions as well. Finally, the coder exceedingly mistook Other for D.

**Table 3:** Performance of the classifier in a semi-inclusive layout.

Classes			Classifier									Human coder									Support, n
		Precision		Recall		F1 score		Accuracy		Precision			Recall		F1 score		Accuracy				
**All**			—^a^		—		—		0.80		—			—		—		0.83		—	
	SG+D^b^	0.76		0.83		0.79		—		0.81			0.82		0.82		—		702		
	D^c^	0.92		0.80		0.86		—		0.88			0.88		0.88		—		1157		
	SG^d^	0.40		0.57		0.47		—		0.61			0.36		0.45		—		69		
	Other	0.16		0.50		0.24		—		0.17			0.30		0.21		—		30		
Weighted score			0.83		0.80		0.81		—		0.84			0.83		0.83		—		—	

^a^Not calculated or not applicable.

^b^SG+D: Screen Gaze and Dialogue.

^c^D: Dialogue.

^d^SG: Screen Gaze.

### Performance in a Fully Inclusive Layout

Table 4 shows the performances of the classifier and the human coder in a fully inclusive layout. The classifier and the coder showed similar accuracy (both equal to 0.86), and there was no significant difference between the accuracy of the classifier and that of the human coder for a fully inclusive layout (*t*_8_=0.43, *P*=0.67).

The classifier and the coder had good F1 scores when classifying D (classifier: 0.92; human coder: 0.93) and SG+D (classifier: 0.83; human coder: 0.80). The classifier performed better than the coder for the SG class (classifier: 0.73; human coder: 0.59), but worse for Other interactions (classifier: 0.42; human coder: 0.52). The D and SG+D classes had the most support(D: 1268/1963, 65%; SG+D: 487/1963, 25%), thus the overall accuracy mainly reflects the performance for these 2 classes.

**Figure 6:**
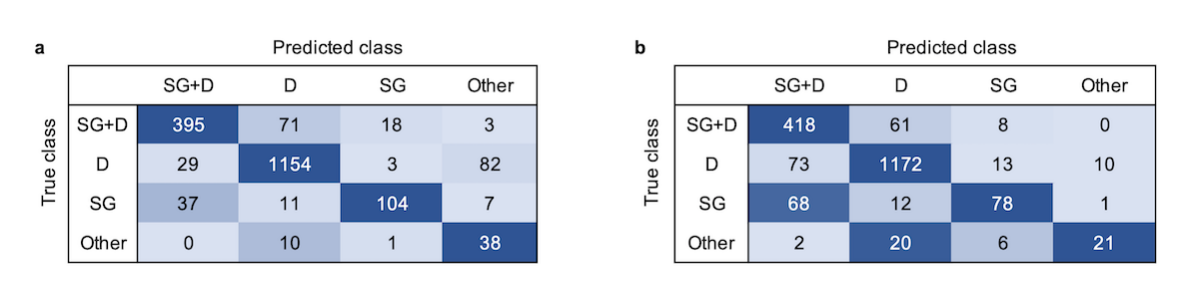
(a) Classifier and (b) human coder confusion matrices for fully inclusive layout. D: Dialogue; SG: Screen Gaze; SG+D: Screen Gaze + Dialogue.

Confusion matrices of the classifier and the human coder for a fully inclusive layout (Figure 6) show similar patterns for the classifier and the coder. Both mistook SG+D for D, SG for SG+D, and Other interactions for D; however, the classifier tended to mostly mistake D for Other interactions, whereas the coder mostly mistook D for SG+D.

**Table 4:** Performance of the classifier in a fully inclusive layout.

Classes		Classifier					Human coder					Support
		Precision	Recall	F1 score	Accuracy	Precision		Recall	F1 score	Accuracy		
**All**		—^a^	—	—	0.86	—		—	—	0.86	—	
	SG+D^b^	0.86	0.81	0.83	—	0.75		0.86	0.80	—	487	
	D^c^	0.93	0.91	0.92	—	0.93		0.92	0.93	—	1268	
	SG^d^	0.83	0.65	0.73	—	0.74		0.49	0.59	—	159	
	Other	0.29	0.78	0.42	—	0.66		0.43	0.52	—	49	
Weighted score		0.88	0.86	0.87	—	0.86		0.86	0.86	—	—	

^a^Not calculated or not applicable.

^b^SG+D: Screen Gaze and Dialogue.

^c^D: Dialogue.

^d^SG: Screen Gaze.

### Transitions Between Doctor-Patient-Computer Interactions

We found that many errors in the classifications (for both the human coder and our classifier) were due to slight time inconsistencies during transitions. To confirm this hypothesis, we conducted an analysis of the transitions between the interactions. Table 5 reports the frequency of each transition in the ground truth data and shows that most transitions happen from D to SG+D and vice versa. We define a transition timing error as a temporal shift of 0.5 to 1 seconds between the ground truth and the classification. We only included transitions that were preceded and followed by a continuous type of interaction for at least 1.5 seconds. Table 6 reports the absolute and relative number of errors that can be attributed to early or late coding of transitions. We think these errors can be overlooked for any practical purpose.

**Table 5:** Transitions in ground truth data.

Transition		Semi-inclusive, n	Fully inclusive, n
**From SG+D^a^**			
	...to D^b^	103	65
	...to SG^c^	10	16
	...to Other	1	0
**From D**			
	...to SG+D	104	67
	...to SG	7	15
	...to Other	10	16
**From SG**			
	...to SG+D	8	13
	...to D	10	17
	...to Other	0	2
**From Other**			
	...to SG+D	1	1
	...to D	10	14
	...to SG	2	4

^a^SG+D: Screen Gaze and Dialogue.

^b^D: Dialogue.

^c^SG: Screen Gaze.

**Table 6:** Transition-related errors.

Layout	Classifier		Human coder	
	Total errors, n	Transition errors, n (%)	Total errors, n	Transition errors, n (%)
Semi-inclusive	400	65 (16.2)	329	73 (22.2)
Fully inclusive	272	45 (16.5)	274	61 (22.3)

## Discussion

### Principal Results

We developed an unobtrusive, inexpensive, and automatic classifier of screen gaze and dialogue combinations in doctor-patient-computer interactions. The classifier was evaluated in 2 clinical layouts, semi-inclusive and fully inclusive, and had a performance similar to that of a human coder with an overall accuracy of 0.83. The proposed classifier is unobtrusive since it does not require additional setup in the clinic and only requires that doctors record video using their computer's internal microphone and camera. The proposed classifier is an inexpensive solution since it is built using open-source tools and takes advantage of the internal camera and microphone built into most available computing devices. Finally, the video can be locally processed, thus reducing the risks of handling private and sensitive data off the clinic's premises, and ensuring that no collateral data are collected and used for purposes other than those initially consented to by the participants.

Both the classifier and the coder had better accuracies in a fully inclusive layout (both equal to 0.86) than in the semi-inclusive layout (classifier: 0.80; human coder: 0.83). The difference in performance can be attributed to the different postures that the doctor maintains when interacting in the 2 clinic layouts. In the fully inclusive layout, the doctor has to rotate their head a full 90 degrees away from the screen in order to gaze at the patient, whereas in semi-inclusive scenarios, the head rotation angle is smaller; therefore, it is easier to make distinctions between the interactions in a fully inclusive layout.

Both the classifier and the coder confused SG+D interactions and D interactions. Some instances occurred when classifying near transitions between interactions. Indeed, our analysis showed that 16.4% of the classifier’s errors (110/672) and 22.2% of the human coder’s errors (134/603) were early or delayed markings of transitions, which can be overlooked in practical use-cases. Unlike the human coder, the classifier tended to mistake D for Other interactions (ie, the doctor is neither looking at the screen nor conversing with the patient). This may be attributed to the fact that human coders overlook small moments of silence and regard them as response offsets that are due to turn-taking in the conversation [55] or lapses that are expected in multiactivity settings [56,57], whereas our classifier classifies them as an absence of dialogue. Here, the classifier presents an advantage since it easily detects moments of silence that are usually overlooked by human coders. This kind of information may be useful to detect conversational dimensions such as hesitant speech [42]. However, for our purposes, this leads the classifier to overestimate the lack of verbal interaction. To counteract this, further rules are needed to identify which moments of silence are part of a conversation and which are not. These rules need to take into consideration the language and the content of the conversation [55,58], other activities that individuals are engaging in while conversing [56,57], and accompanying nonverbal behavior such as nodding and gaze [55].

As a collateral result, our experiment also confirmed the findings of previous work that highlighted the effect of the clinic layout on doctor-patient-computer interactions [59,60]. Fully inclusive videos contained more D (64% versus 59%) and SG interactions (8% versus 3.5%) and fewer SG+D interactions (25% versus 36%) than those in semi-inclusive videos. In a fully inclusive layout, the doctor has to choose whether to face the computer or their patient, whereas a semi-inclusive layout allows the doctors to maintain a conversation with their patients while looking at the computer screen.

### Use Cases

#### Functionality

Because health communication researchers study various complex behaviors, the coding process is far more complex than a binary classification of screen gaze and dialogue over time. However, the proposed classifier could contribute to reducing the cost of future studies since gaze and dialogue are part of the behaviors that health communication researchers and medical informaticians are often interested in quantifying to examine the relationship between computer use, clinic and tool design, and physician-patient interactions [3,5,19,28,61-65]. Moreover, by detecting these behaviors over short intervals of time, more complex behaviors and patterns could be inferred as shown in our results on transitions between interactions. Therefore, the classifier can be directly used to monitor screen gaze and dialogue or extended and combined with other tools or processes to examine complex interactions in clinical settings.

#### For Researchers

The proposed tool can be used to study the effects of screen gaze and dialogue combinations in doctor-patient-computer interactions on quality of care and health outcomes. Currently, conducting this type of study would require (1) recording a video of the consultation, (2) transferring the video outside of the clinic, (3) manual coding of screen gaze and presence of dialogue in the video, and (4) assessing the specific outcome. This process can be facilitated by our classifier, which eliminates the need for the second and third steps.

The classifier can also be used in clinics to examine the effect of external factors on screen gaze and dialogue combinations in patient-doctor-computer interactions such as sociodemographic characteristics, clinic layout, and modifications of electronic medical record system’s design. Non–self-reported large-scale studies of this kind would be nearly impossible to conduct using current methods and tools.

In addition, the gaze classifier can be used in conjunction with commonly used interaction analysis coding systems, such as the Roter interaction analysis system [42], that do not systematically account for nonverbal behaviors [66]. This would provide useful data for studies examining provider-patient communication in the presence of a computer.

#### For Practicing Physicians

Since the processing of the video can happen in real time, tools that allow physicians to *reflect in action* [67] can be created. The classifier can be used to create tools that allow physicians to conduct autoethnographies and reflect on their interactions with the patient, or on the role of technology in their care practice [68]. This would allow them to adapt their behavior and level of attention based on feedback. Similar concepts were proposed by Liu et al [69] and Faucett et al [51], who described tools that provide feedback to clinicians about their verbal and nonverbal communication behaviors during online consultations. Their studies highlighted the usefulness of summative [70] and real-time self-reflection tools and the need to design real-time feedback in a way that minimizes intrusiveness and ensure that it does not create extra distractions [51].

#### For Medical Educators and Students

Medical educators and students can use the classifier to teach and learn the best practices of doctor-patient interactions. The tool could be expanded to detect different interaction categories (eg, listening/ignoring; confronting/avoiding [71,72]) and used during practical learning sessions to provide students with formative feedback [69].

### Limitations and Future Work

The first limitation of the classifier is its applicability to certain clinic layouts. Our evaluation explored 2 clinical scenarios: semi-inclusive and fully inclusive. We did not explore exclusive scenarios, even though these scenarios might be encountered in real-world clinical settings. With the proposed classifier, detecting the doctor's computer gaze would not be possible in a fully exclusive scenario since the classifier considers the doctor's head turn to estimate her gaze. We consider this limitation acceptable since inclusive scenarios are already commonplace [25], and we expect an increase in their prevalence to support technology-mediated information-sharing between clinicians and patients. Indeed, previous studies [31,73,74] have shown that clinicians use their computer screens as tools to share information with their patients; therefore, the doctor may turn the screen, along with the camera, toward the patient. If this interaction happens during an ongoing conversation, our tool may classify it as a dialogue between doctor and patient, but the fact that this doctor-patient interaction is mediated by the computer would not be highlighted. Further improvements are needed to detect scenarios where both clinician and patient are interacting with the computer at the same time.

Our work also assumes that clinicians use their computers during consultations, which is not always the case, especially in secondary care settings. Moreover, the classifier is built and evaluated around the premise that the only people in the clinic are the doctor and the patient and that the only screen is the doctor's computer screen. However, it is possible that patients are accompanied by their family members, friends, or partners [75] and that multiple health care staff are involved in the care of 1 patient and present during the consultation. In addition, extra screens might be installed inside clinics to engage the patient in their care and offer them an easy and clear view of their data. These screens may affect the doctor's behavior in various ways; for example, they might use this screen as an explanation support tool while they converse with the patient or even as their main computer screen. Therefore, another limitation of this work is its nonapplicability in scenarios that include patient screens and stakeholders other than the doctor and patient.

Furthermore, the classifier does not allow us to identify the speaker's identity or the content of the doctor-patient dialogue. Therefore, the classifier does not currently support conversation analysis. To identify the speaker's identity, we would have to perform accurate speaker diarization, a hard goal to achieve especially using a single channel for audio recording and without prior training. Advancements in speaker diarization techniques may render this task feasible in the near future [76]. To identify the content of the dialogue, automatic speech recognition solutions can be used. Though automatic speech recognition solutions have become more robust in the last decade, the performance of automatic speech recognition engines remains limited when applied to conversational clinical speech [77]. Future work could explore the feasibility of automatic conversation analysis in doctor-patient-computer interactions through the application of novel speaker diarization and automatic speech recognition tools.

In other respects, although the direction of the head could be considered a proxy for the direction of attention, the head only communicates short-term attention [78,79]. Pearce et al classified physicians as unipolar, those who maintain the lower pole of their body facing the computer, or bipolar, those who repeatedly alternate the orientation of their lower pole between the computer and the patient [34]. Unipolar physicians experience situations where their body segments are not aligned, also referred to as body torque. Body torque communicates an instability of attention where the most strongly projected resolution involves the upper body getting realigned with the lower body. This means that the orientation of the torso communicates longer-term attention than head orientation, and the orientation of the legs communicates longer-term attention than torso orientation and head orientation [79]. Therefore, to examine the attention of a physician in a clinical scenario, we also need to examine the orientation of their torso and lower body. Future work can use the same pose estimation approach to monitor the direction of the clinician’s torso. This is possible because the shoulders and the torso of the clinician are usually visible to their computer’s camera; however, monitoring the lower part of the body would require setting up extra cameras in the clinic.

Finally, our classifier is model-driven as it derives its decisions from the explicit rules that we set. To be able to classify interactions that do not fit strictly into our specified rules, the classifier has to be driven by data or perhaps be a system that combines model-driven and data-driven logic. Our future work will include collecting and annotating more videos of consultations in order to create a data-driven classifier.

### Conclusions

To facilitate human-computer and human-human interaction studies in clinical settings, we presented a computational ethnography tool—an automatic unobtrusive classifier of gaze and dialogue combinations in doctor-patient-computer interactions. The classifier only requires that the doctor record video using their computer’s internal camera and microphone. Our evaluation showed that the classifier's performance was similar to that of a human coder when classifying 3 combinations of screen gaze and dialogue in doctor-patient-computer interactions.

## Abbreviations

D: Dialogue
SG: Screen Gaze
SG+D: Screen Gaze and Dialogue
